# Recent Development in Therapeutic Cardiac Patches

**DOI:** 10.3389/fcvm.2020.610364

**Published:** 2020-11-27

**Authors:** Xuan Mei, Ke Cheng

**Affiliations:** ^1^Department of Molecular Biomedical Sciences and Comparative Medicine Institute, North Carolina State University, Raleigh, NC, United States; ^2^Joint Department of Biomedical Engineering, University of North Carolina at Chapel Hill and North Carolina State University, Raleigh, NC, United States

**Keywords:** biomaterials, cardiac patch, myocardial infarction, cell therapy, cardiac tissue regeneration

## Abstract

For the past decades, heart diseases remain the leading cause of death worldwide. In the adult mammalian heart, damaged cardiomyocytes will be replaced by non-contractile fibrotic scar tissues due to the poor regenerative ability of heart, causing heart failure subsequently. The development of tissue engineering has launched a new medical innovation for heart regeneration. As one of the most outstanding technology, cardiac patches hold the potential to restore cardiac function clinically. Consisted of two components: therapeutic ingredients and substrate scaffolds, the fabrication of cardiac patches requires both advanced bioactive molecules and biomaterials. In this review, we will present the most state-of-the-art cardiac patches and analysis their compositional details. The therapeutic ingredients will be discussed from cell sources to bioactive molecules. In the meanwhile, the recent advances to obtain scaffold biomaterials will be highlighted, including synthetic and natural materials. Also, we have focused on the challenges and potential strategies to fabricate clinically applicable cardiac patches.

## Introduction

Heart disease remains a leading problem threatening millions of people worldwide (1–3). Due to the lack of regeneration ability, cardiomyocytes (CMs) in adult mammalian heart can hardly recover from ischemic injuries, like myocardial infarction (MI) (4–7). Suffered from such an irreversible cardiac muscle death, CMs will be gradually replaced by fibrotic scar tissues (8–10). The loss of the contractile capacity leads to the dysfunction of heart and causes heart failure eventually (11, 12). Thus, it is still a challenge to explore novel therapeutic methods for myocardium regeneration.

**Figure 1:**
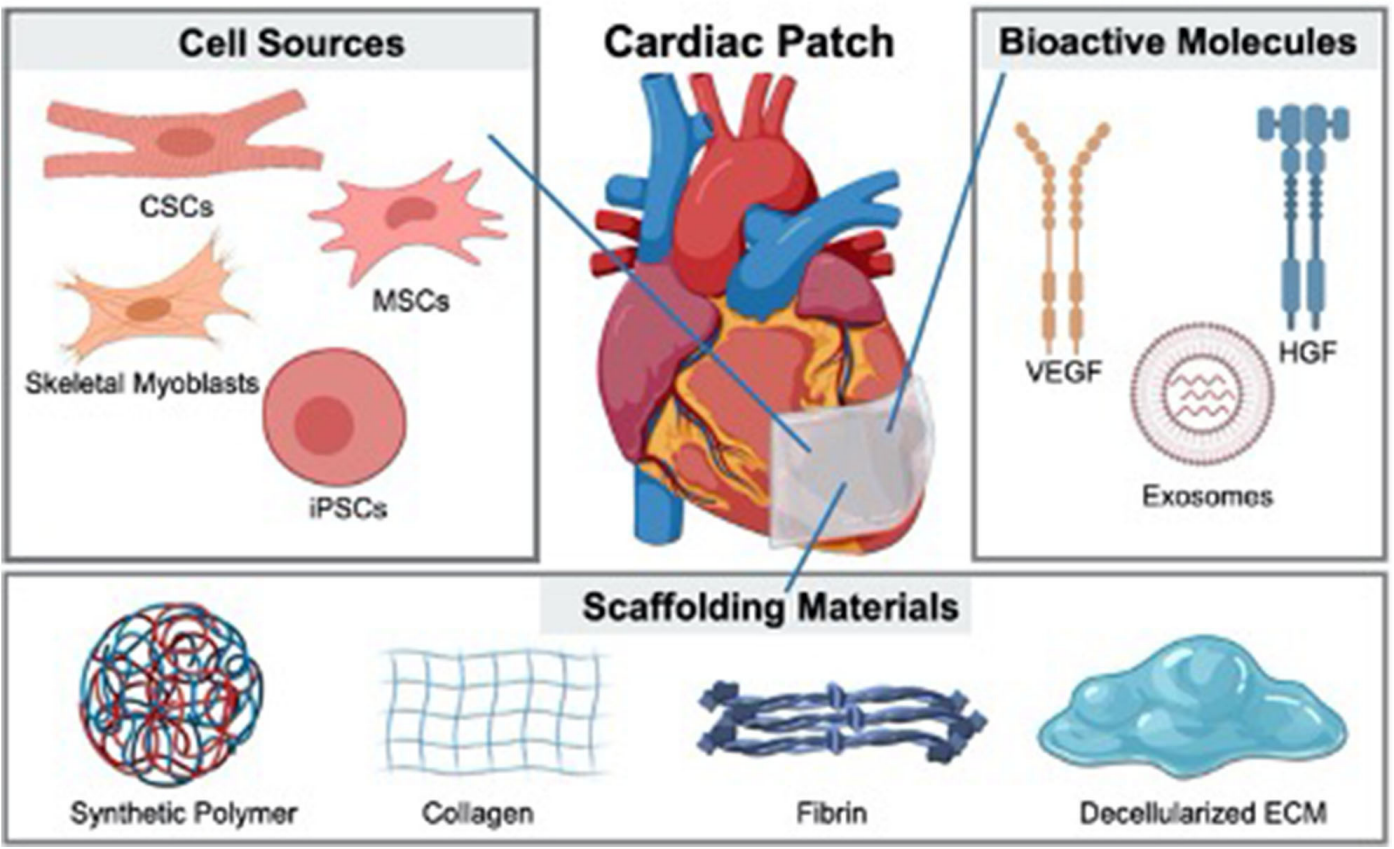
Cardiac patches fabricated from different types of cells and bioactive molecules with various scaffolding materials.

Preclinical studies have demonstrated the therapeutic performance of several approaches to treat MI, such as the injection of stem cells (13, 14), genes (15, 16) and growth factors (17). However, these therapeutics always suffer from low stability and short half-lifetime. Hence, delivery methods are highly demanded to achieve better therapeutic performance. In virtue of the engineered biomaterials, cardiac patches show promising potential in promoting cardiac function (18, 19). The component of a cardiac patch can be simply divided into two parts: substrate and therapeutic ingredients (20). With the development of fabrication technologies, more and more cardiac patches with excellent therapeutic performance have been developed (21) (Figure 1). The therapeutic ingredients for cardiac patches range from cells (such as skeletal myoblasts, mesenchymal stem cells and human pluripotent stem cells) to bioactive molecules (including growth factors, microRNA and extracellular vesicles) (22–24). A large number of biomaterials used to fabricate cardiac patches have emerged during the last decade (25–28). Whether natural or synthesized, these scaffolding materials offer a suitable environment for therapeutic ingredients (29–31). Polymers are the most used materials for cardiac patch fabrication (32, 33). Some patches are generated from *in vivo* sources like collagen (34), fibrin (35), decellularized ECM (36) and even cell sheets (37), providing outstanding biocompatibility compared to synthesized materials (38). In this review, both the therapeutic ingredients and biomaterials will be discussed (Table 1), but topics such as disease modeling (58), bioreactor stimulation (59), microphysiologic systems (60) will not be included. In addition, we will focus on the limitations of current cardiac patches to clinical application.

**Table 1:** Representative studies of cardiac patches in recent years.

**Cell/Therapeutics type**	**Scaffolding material type**	**Animal model**	**Major findings**	**References**
Cardiac stromal cells	PVA microneedle patch	Rat and porcine	Provide channels for the communication between the patch and the host myocardium	(39)
Synthetic cells	ECM	Rat and porcine	Long term storage ability and clinically feasible	(40)
Mesenchymal stem cells	ECM/SF with Au NPs	Rat	Improve the cell proliferation and migration	(41)
	Collagen with VEGF and bFGF	Rat	Pro-angiogenic, anti-apoptotic and -inflammatory	(42)
	PPy coated PCL	Rat	Accommodate the strains and stresses of the human myocardium	(43)
	MSC-fibrin and collagen	Rat	Improve the retention and reparative functions of MSCs	(44)
	ECM	Rat	*In vivo* primed hepatocyte growth factor MSCs for better therapeutic performance	(45)
	PEGDMA	Mouse	Gel patch with microchannels for better attachment	(46)
Human pluripotent stem cells	ECM	Rat	The first non-supplemented bioink for 3D printing	(47)
	Fibrin scaffold	Porcine	Clinically relevant size (4 × 2 cm × 1.25 mm)	(48)
	Biomaterial-free	Rat	3D-bioprinted biomaterial-free cardiac tissue patch	(49)
	Fibrin and human Microvessel patch	Mouse	Rapidly perfused *in vivo*	(50)
	A blended fibrin and collagen scaffold	PDMS molds	An analytical method to better understand cell-scaffold interactions	(51)
	Cell spheroids	Rat	Biomaterial-free cardiac tissues created by a novel net mold system	(52)
	Scaffold free	*In vitro*	A scalable method to fabricate scaffold-free human cardiac tissue patches	(53)
	PGS	Rat	Revealed the advantages of PGS for stem cell-based cardiac regeneration	(54)
SDF-1	Electroactive polymers	*In vitro*	Freestanding electronics integrated into a 3D nanocomposite scaffold	(31)
HGF and IGF-1	Collagen scaffold	*In vitro*	Promote the expansion of CSCs	(55)
Exosomes	PGN hydrogel	Rat	Heart function improved with human umbilical cord MSCs derived exosomes	(56)
	Shear-thinning gel	Rat	Enhanced efficacy of exosome-mediated myocardial preservation	(39)
	Silk fibroin hydrogel	Mouse	Blood perfusion promoted by miR-675 contained exosomes	(57)

## Cell Sources

The first type of cell reported for heart regeneration was skeletal myoblasts (61, 62). Up to now, the functional benefits of skeletal myoblasts to treat ischemic heart diseases have been proved in clinical trials (63, 64). Since then, cell therapy for cardiac regeneration has attracted wide interest (65). More and more types of cells have been studied, including cardiac stem/stromal cells (66), mesenchymal stem cells (MSCs) (67) and human pluripotent stem cells (68). To enhance the cellular retention and survival ratio, cell based cardiac patches have been developed (69–72).

### Cardiac Stem/Stromal Cells

Cardiac stem/stromal cells, including cardiosphere-derived cells (CDCs) (73), stem cell antigen-1^+^ (Sca1^+^) cells (74) and lslet-1^+^ (Isl-1^+^) cells (75), have shown preclinical and clinical evidence in ischemic tissue preservation and anti-remodeling. Nowadays, the transplantation of CSCs for heart repairing has been achieved clinically, however, the adverse immunoreaction severely hampers the treatment performance (76, 77). One of the strategies to enhance the safety during CSCs transplantation is to introduce nanogel for encapsulation. In a recent work by Tang et al. (78), human CSCs (hCSCs) were encapsulated in thermosensitive P(NIPAM-AA) nanogel. It was found that with the injection of nanogel, barely systemic inflammation or local T cell infiltrations was elicited by hCSCs in immunocompetent mice. Compared to xenogeneic hCSCs injection in saline, these nanogel-encapsulated hCSCs exhibited largely reduced adverse effect, while still preserving cardiac function and reducing scar sizes in mouse and pig models. Another limitation of CSCs therapy in cardiac diseases is the low cellular retention and survival ratio (79). Cardiac patch provides an excellent platform for cell engraftment improvement. For example, a vascularized cardiac patch was recently developed, which shows promising benefits to treat ischemic heart injury (80). This patch was fabricated by encapsulating the biomimetic microvessels (BMVs) and CSCs in a fibrin gel. As tested in a rat MI model, the myocardial capillary density was improved significantly after BMV–CSC patch therapy, which can be ascribed to the paracrine factors release.

### Mesenchymal Stem Cells

With the strong ability of differentiation and immunoprivileged nature, MSCs have been the most studied cell type for cardiac cell therapy in clinical trials (81, 82). MSCs can be derived from various organs and tissues, especially bone marrow and adipose tissue (83, 84). According to current researches, the therapeutic effects of MSCs are achieved through the secretion of paracrine effectors (such as growth factors) (85). To achieve better treatment, various methods have been developed to extend the therapeutic potential of current MSCs (86). Primed MSCs through genetically engineered hepatocyte growth factor–expressing MSCs (HGF-eMSCs) was developed by Park et al. (45), showing largely improved vasculogenic ability and enhanced cell viability. After encapsulated in a cardiac patch, this kind of MSCs promoted vascular regeneration and repaired cardiac function within MI hearts. Another issue for the clinical use of MSCs is their restricted retention ability after transplantation into failing hearts. Recently, a minimally invasive approach was developed by Lee et al. (87), which utilized an array of microneedles with an outer shell [poly(lactic-co-glycolic) acid] and an internal gelatin methacryloyl (GelMA)-MSC mixture (GMM). The mechanical strength and the regenerative ability were demonstrated *in vitro* and *in vivo*. Apart from bone marrow, other tissues also exhibit MSCs derived potential, including adipose, placenta, and amnion (88). Cells derived from different sources present diverse treatment performance after transplantation into MI animals (89).

### Pluripotent Stem Cells

Human pluripotent stem cells, including embryonic stem cells (ESCs) and induced-pluripotent stem cells (iPSCs), have been proved to generate various types of cells in the area of regeneration (90). ESCs, derived from inner cell mass of pre-implantation blastocyst, show the potential of differentiation into cardiomyocytes (91). Compared to adult cardiomyocytes, these differentiated cardiomyocytes have similar physiological characteristics and can beat spontaneously (92). ESCs can also differentiate into other types of heart cells, such as endothelium and vascular smooth muscle (93). Moreover, these cells show excellent ability in releasing paracrine factors for heart repair, including growth factors, antifibrotic and antiapoptotic (94). Another technology to fabricate ESCs is parthenogenesis-asexual, which has successfully achieved in mice and non-human primates through chemically stimulated (95).

Similar to the chemical stimulation process in producing ESCs, the generation of iPSCs can be achieved by introducing different transcriptional regulators (such as Oct4, Sox2, Klf4 and Myc) (96). Taking the advantage of derived patients' cells, the utilization of iPSCs in treating heart diseases benefits a lot from immunological aspect (97). The clinical potential of human iPSCs (hiPSCs) derived cells encapsulated patches have been explored. By suspending three different cardiac cells (cardiomyocytes, smooth-muscle cells, and endothelial cells) derived from hiPSCs in fibrin scaffold, a clinically relevant size (4 × 2 cm × 1.25 mm) human cardiac muscle patches (hCMPs) was fabricated (48). It was found that the infarct region was significantly reduced after the transplantation of this patch. Because of the reduction in LV wall stress, cardiac function was largely restored while the arrhythmogenicity showed little change. The safety of hiPSCs in clinical trial has been confirmed, while the enhancement of therapeutic efficacy is still on the way (98, 99). A dual approach was explored in recent study (100). Cardiomyocytes derived from human induced pluripotent stem cells (hiPSC-CMs) along with a hMSCs patch were applied in the meantime in MI rat model. Since the hMSCs patch provided a microenvironment for hiPSC-CMs to survive, the cardiac function of rat was better restored then hiPSC-CMs along. Because of the ethical controversy and immunological issues, it has been reported that iPSCs takes more advantages in heart regeneration than ESCs (101). To better understand the bioactivity of iPSCs, more specific characterization like genetic programming is highly demanded.

## Acellular Patch

Instead of the direct interact of cells with host cardiomyocytes, paracrine factors released by cells play a key role in heart repair (102). Among these factors, growth factors (GFs), extracellular vesicles (EVs) and microRNAs are mostly studied (103). Different from cellular patches, acellular patches integrated with paracrine factors exhibit more direct therapeutic effects (104). In addition, patches fabricated with only biomaterials also show passive restrained ability toward injured myocardium (105).

### Growth Factors

As one of the paracrine effectors, the significant role of growth factors in multiple cellular processes have been determined. Accumulating evidence supports that growth factors, such as vascular endothelial growth factor (VEGF), hepatocyte growth factor (HGF) and insulin-like growth factor-1 (IGF-1), regulate the growth, survival and migration of cardiomyocytes (106). To protect growth factors (mostly proteins) during the delivery process as well as target the tissue site, growth factors encapsulated cardiac patches have attracted tremendous attention (107). Before clinical trials, the efficiency of growth factors integrated cardiac patches have been evaluated via various animal models. For example, a growth factor embedded nanofibrous patch was developed and tested in a rabbit MI model (107). With the rising death rate of cardiac diseases, the on-demand release of therapeutic factors shows significant in clinical treatment. Cardiac patches integrated with complex electronics were fabricated, allowing the on demand releasing of growth factors (31). With the electronics, this patch also performed cellular electrical activities recording ability, facilitating the electrically triggered cell contraction. Cardiac patches provide a protection for growth factors from easy elimination. Due to the pleiotropic functions of growth factors (108), local delivery through cardiac patch shows significance to avoid unexpected adverse effects.

### Extracellular Vesicles

Extracellular vesicles (EVs) are miniscule vesicles with a diameter between 30 and 150 nm (109). It is highlighted that EVs play a key role in cell communication, regulating various intercellular activities (110). The certain EVs markers are still under exploration because of their complex overlapping surface properties. Based on the surface proteins, EVs can be generally divided into three types: apoptotic bodies, microvesicles, and exosomes (111).

Among all the EVs, exosomes secreted by stem cells have been studied extensively (112). Exosomes are phospholipid bilayer-enclosed vesicles with contents inside, such as proteins, mRNA and miRNA (113). Recent studies have illustrated that it is paracrine factors rather than MSCs themselves contribute to treating cardiovascular diseases (114). Moreover, lots of pre-clinical evidence confirmed the cardiac function restored ability through exosomes that extracted from MSC conditioned media, demonstrating the potential cardiac repair performance of exosomes (115). In addition to MSCs, exosomes secreted by other cell sources, including iPSCs derived CMs, hESCs derived CMs and monocyte, also help with cardiac repairing. For example, a hydrogel patch encapsulated with EVs derived from iPSCs derived CMs was developed by Liu et al. (104), which allowed continuous treatment during the whole phase of heart injury. In another work, PGSA-g-EG polymer was investigated for the fabrication of cardiac patch (116). With the loading of EVs, this adhesive cardiac patch exhibited a controlled release behavior for more than 14 days. As evidenced by the miRNA analysis, exosomes secreted by iPSCs derived CMs and hESCs derived CMs have the same functional miRNAs for heart repair (117). Because of the short half-life, current therapies utilizing exosomes lack long-term effect. Although recent studies have developed various delivery methods for exosomes (118), explorations should still focus on fabrication technology to meet clinical demands.

### MicroRNAs

MicroRNAs (miR) are a kind of single-stranded RNA with a small number of nucleotides (usually 20–25) (119). Previous researches have shown that miRs play an important role in regulating downstream pathways of messenger RNA through RNA interference, indicating effective cell modification ability (120). In particular, both the survival and apoptosis of cardiomyocyte are under the control of the miR network, such as miR-24, -199a, and -590 (121). Thus, miR related therapy for myocardial infarction (MI) has been identified as a promising treatment method (24). Despite the feasibility of miR therapy, several issues need to be addressed before further practical studies. Since naked miRs are unstable during *in vivo* circulation, the leading challenge would be how to deliver miRs to the targeted site with enhanced retention (122).

To overcome these obstacles, several methods have been investigated, including chemical modification and carrier development (123). For example, a recent study showed that with the assistance of a tissue-engineered three-dimensional (3D) culture environment, the reprogramming efficiency of miRs from cardiac fibroblasts into functional cardiomyocytes was enhanced (124). Such an improvement was confirmed from both gene and protein levels by the assessment of cardiac differentiation markers, which provided a fundamental basis for the fabrication of cardiac patch with miRs. Given that several human miRs, such as hsa-miR-199a-3p, exhibited therapeutic potential in cardiac regeneration, a pig MI model was constructed for more clinically relevant research (15). In this 1-month study, the de-differentiation and proliferation of cardiomyocyte were observed, indicating the expression of human microRNA-199a contributed to restoring cardiac function. However, unexpected sudden arrhythmic death occurred among treated pigs, which required further careful control on the dosage. The delivery of miRs with cardiac patches is appealing for cardiac restoration, but more detailed studies are required to better understand this emerging technology.

### Acellular Patch Without Biomolecules

In spite of providing a pathway for cellular or biomolecule delivery, cardiac patches fabricated with only acellular biomaterial matrices have shown potential to treat MI (125). Served as mechanical-structural supports, such cardiac patches without any external therapeutic agents exhibited passively restraining ability toward infarcted ventricle, protecting left ventricular from remodeling and dilation (126). To understand the mechanisms as well as maximize the therapeutic efficiency, a simulation-guided strategy was carried out recently (21). In this study, finite-element simulations were applied to simulate the remodeling of left ventricular, which accounts for the balanced material properties between fluid and solid. Benefit from such design, a viscoelastic adhesive patch was fabricated, which not only restrained dilatation but also restored the cardiac function after MI. To match the mechanical properties of heart tissue, technologies such as excimer laser microablation was applied for the fabrication of mechanical support cardiac patch (127). By virtue of such micropattern strategy, the patch was determined with a negative Poisson's ratio, showing priority in conforming to the mechanics of the heart. Although the design of such matrices only cardiac patches is to provide mechanical support to reduce pathological cardiac remodeling, their contributes to providing a favorable physiological environment for biomolecule delivery cannot be ignored (105).

## Materials for Patch Fabrication

The exploration of materials used to fabricate cardiac patches never stops. Due to the unstable bioactivity of cells or other biological molecules, a substrate is highly needed to provide cellular microenvironment as well as enough mechanical support (128). Some key points should be taken into consideration when select suitable materials, such as biocompatibility, biodegradability and mechanical strength (129). In the meantime, materials whether synthetic or natural, must be deliberated carefully to meet the demands of clinical applicability (130), such as easy to acquire and long-term storage. In this section, biomaterials for cardiac patch fabrication will be discussed from synthetic to natural ones.

### Synthetic Materials

Taking advantages of reproducible synthesis processes, synthetic materials with stable physical and mechanical properties have shown potential to meet clinical requirements (131). A lot of synthetic materials for tissue engineering have been well-studied, such as polymer poly(vinyl alcohol) (PVA), poly(lactic-co-glycolic) acid (PLGA), poly-(L-lactic) acid (PLLA) and polyurethanes (PU) (132).

With the strong mechanical properties, it is possible to make a linker between cells and host myocardium through a synthetic polymer patch (133). Currently, how to deliver secreted factors efficiently to MI region remains a challenge in cardiac stem cell therapy. Microneedle patch provides such an opportunity for drug delivery. Recently, a cardiac stromal cell-encapsulated microneedle patch (MN-CSCs) was fabricated through micromolding approach, using biocompatible PVA (39). Microneedle played a role as the channels between cells and the host myocardium, from which cells got sufficient nutrients to survive and released more paracrine factors to restore the heart function.

Due to the strong mechanical strength, acellular epicardial patches also show excellent therapeutic efficacy to help rebuilt damaged cardiac tissues. A viscoelastic adhesive patch was developed, exhibiting LV remodeling reversing ability in both acute and subacute MI rats model (21). This patch was made from ionically crosslinked transparent hydrogel with a low dynamic modulus. Through finite-element simulations, this acellular epicardial patch was designed at the “gel point,” contributing to the balance between fluid and solid properties. Without biomolecules or cells, cardiac patches made from synthetic materials have priorities than natural materials for clinical application, such as long-term storage, stable quality and easy manufacturing process.

### Natural Materials

Different from synthetic materials, natural materials such as collagen, fibrin, alginate, hyaluronic acid, gelatin, and decellularized extra-cellular matrix (ECM) show superior biocompatibility (134). Whether derived from *in vivo* sources or naturally occurring, these materials retain the structure to mimic originally cellular microenvironment. Notably, materials generated from biological sources offer extra protection for therapeutics to escape from immune inflammation, allowing improved therapeutic function (135).

Collagen is the most widely used natural material for cardiac patch fabrication, which mainly exists in cardiac ECM. Due to the minimal antigenicity and chemotactic properties, collagen can provide a tissue-like environment for cells (136). The latest technology to fabricate collagen patches including electrospinning, which requires electrically charging. Different cells can be seeded into electrospun nanofibrous collagen scaffolds, such as iPSCs and MSCs (39). Benefit from paracrine signaling and force transmission, collagen scaffold patches show potential therapeutic performance in treating both MI and dilated cardiomyopathy (DCM). The development of conductive collagen cardiac patch has become a tendency. With the addition of conductive components, such as carbon nanotubes, metal nanoparticles and graphene oxide, the online monitoring of tissue statues can be achieved (127). Other *in vivo* source natural materials, like fibrin, HA and alginate, also exhibit therapeutic potential for clinical studies.

Currently, the most studied biomaterials for cardiac patch fabrication is decellularized ECM (137). Either derived from cardiac sources (such as myocardium and pericardium) or non-cardiac sources (small intestinal submucosa and urinary bladder matrix), decellularized ECM provides an ideal environment to support cardiac restore processes (138). In addition, recent studies also demonstrated the vital roles of decellularized ECM in cardiac repair. Inspired by the excellent regeneration ability of neonatal mouse heart, researchers found that one of the proteins in neonatal extracellular matrix named agrin, played a key role in promoting heart regeneration (139). This kind of protein helped with the disassembly of the dystrophin–glycoprotein complex, thus promoted the division of cardiomyocytes *in vitro*. For cardiac stem therapy with patches, the difficulty in cell viability retaining becomes the limitation for clinical trials. To solve this problem, a strategy using synthetic cardiac stromal cells (synCSCs) was generated to fabricate an off-the-shelf therapeutic cardiac patch (40). Through the encapsulating of synCSCs on to the decellularized ECM, the potency of this fully acellular artificial cardiac patch (artCP) was well-maintained for a long time. The cardiac repair function of the artCP was confirmed in a rat MI model. Furthermore, the clinical potential of this artCP was revealed in a porcine model with MI.

The strong physical properties of decellularized ECM allow the application of novel technologies during fabrication process, such as 3D Printing. For cardiac tissue engineering, immunological problem remains an unmet gap for patients. Recently, personalized bioink for 3D printing was developed from ECM hydrogel (47). Since this ECM was generated from the patient's personalized omental tissue, the biocompatibility of this bioink was guaranteed. With the addition of two different types of cells, cardiac patch with enhanced vascularization ability was printed. Moreover, cellularized hearts with a natural blood vessel architecture was printed via this kind of bioink. Such technology extended the further potential of ECM for tissue engineering in personalized therapy.

### Scaffold-Less Cardiac Patches

Patches made from scaffold materials have shown priority in improving cell engraftment, however, problems remain in the cause of undesirable arrhythmogenicity and immune rejection toward xenogeneic materials (140). To avoid these drawbacks, the strategy to develop scaffold-less cardiac patches has been developed. As one of the most promising scaffold-less cardiac patches, cell sheets have demonstrated some advantages, including a more biomimetic microenvironment and better cell-cell communication (37). Currently, the fabrication of cell sheets involves a specially coated cell culture dish, which is covered with a temperature-responsive polymer named poly-N-isopropylacrylamide (141). When temperature changes from 37 to 20°C, the surface of this polymer will turn from hydrophobic to hydrophilic because of the conformation transformation. Cells only adhere to the hydrophobic surface and detach from the hydrophilic surface. During the fabrication process, cells are firstly cultured under 37°C until the formation of a confluent film. Afterwards, cells are placed at 20°C until detached spontaneously. Eventually, the cell sheets can be obtained in the upper layer of the media.

The first in-man study of cell sheets was reported in 2012 (142), in which a male with idiopathic dilated cardiomyopathy received the transplantation of cell sheets made from his skeletal myoblasts. Showing the treatment efficiency and safety, cell sheets made from different cell types have been reported, like myoblasts, cardiomyocytes and stem cells. Recent studies have demonstrated the advantages of adipose-derived stem cells (ASCs) in heart regeneration (143). The transplantation of ASCs sheet was developed, which showed restoration ability toward the injured heart. It has been proved that the enhanced secretion of VEGF induced by norepinephrine was the functional process for ASCs sheet therapy. In addition to the secretion of paracrine factors, heart therapy via cell sheets also benefits from the prevention of arrhythmogenicity. It was demonstrated for the first time by Suzuki et al., that skeletal myoblasts (SMBs) sheets not only restored heart function but also prevented ventricular arrhythmias by maintaining the regular electrical conductance (144). To better understand the therapeutic effects of cell sheets, the mechanism studies remain urgent. In addition, it is necessary to seek novel fabrication technology to meet the complicated requirement of clinical trials.

## Challenges and Further Direction

Although cardiac patches have shown promising performance in pre-clinical studies in cardiac repair, problems still remain before their clinical implementation (145). Therapeutic integrates benefit a lot from cardiac patches with enhanced delivery efficiency, however, the implementation of most cardiac patches requires open-chest surgery. As known to all, patients with MI may be not strong enough to recover from the surgical caused damage and inflammation, which brings panic to patients with psychological anxiety (146). Thus, minimally invasive delivery of cardiac patches is highly needed. Not only the implantation method should be improved, novel fabrication technology as well as materials should be the strong back support. More precious technologies, such as 3D printing and photoetching, are highly recommended for patch fabrication (147). Materials with shape memory ability and stronger mechanical property would be the next generation of biomaterials for cardiac patches.

Although it has been confirmed in animal models, the biocompatibility of current cardiac patches is still far below satisfaction and unable to meet the clinical requirement (148). On the one hand, the integration ability of patches with host myocardium is important in enhancing therapeutic efficiency, like improving cardiomyogenesis and angiogenesis at injured heart. On the other hand, it should be highly addressed that the tissue adhesion after cardiac patch transplantation appears commonly, leading to severe side effects (149). It is an immune response since patches are still foreign constituents. This would be the vital concern before cardiac patches can be applied clinically. Currently, researchers have discovered that surface modification will largely reduce the tissue adhesion (150), which may be good candidates for polymer cardiac patches. The biodegradation should also be taken into consideration, as immune rejection will last unless the patches can be degraded after treatment (151). Such improvement requires the biodegradable of materials themselves, while having little impact on therapeutic period.

In addition, long-term storage is necessary for clinical application. Because of the special requirements for cell culture, current cell therapy with cardiac patches are time-consuming and mostly can only be achieved in laboratory (152). How to retain the viability and functionality of cells remains a block for the large-scale production of such therapeutic patches. Although various studies have found that cell retention and engraftment are enhanced to some extent with cardiac patches (153), the therapeutic efficiency is still far below clinical requirement. Artificial materials have a similar function to cells that are highly needed, which requires more mechanism studies of cell therapy for heart regeneration. In conclusion, the development of cardiac patches paves the way for cardiac repair and gives inspiration for heart regeneration.

## Author Contributions

XM wrote the text of this review paper with guidance from KC. Both authors have reviewed the final version and approve of the content in this manuscript.

## Conflict of Interest

The authors declare that the research was conducted in the absence of any commercial or financial relationships that could be construed as a potential conflict of interest.
